# Supplementary material for: Experimental study on the effect of Ivermectin on cattle dung faunas in Eastern Ethiopia
Source: PLoS One. 2025 Apr 16;20(4):e0320867. doi: 10.1371/journal.pone.0320867 (PMC12002493; doi:10.1371/journal.pone.0320867)
Supplement: Attachment — Submitted filename: Response to Reviewers.docx [file pone.0320867.s002.docx]

**Response to Reviewers ’comments**

**Manuscript title: Experimental study on the effect of Ivermectin on cattle dung fauna sin Eastern Ethiopia**

| **Line** | **Comments given by Reviewer #1** | **My responses** |
| --- | --- | --- |
| L1 | Add the country of the study | Thank you for your comments, which I fully accept. I have added. |
| L22 | Change “can influence negatively plant | Accepted the comment as it is. |
| L26 | Keywords must be to differ from those in the title | Thank you for your comments. The keywords were selected based on their frequent occurrence in the manuscript. However, a few modifications have been made in response to the reviewers' feedback. Keywords: *Beetles, Cattle, Dung pats, Ivermectin, soil fertility.* |
| L40,51,222: | Add a comma before and | Corrected |
| L48, 414 | Species name must be italic | Corrected, but 48 and 419. |
| L49, 51, 448, 454 | add a space between the last word and the brackets of the reference | Corrected |
| L97, 417: | add Tovar et al. (2023) Diversity | added |
| L173: | add a reference to support this statement. | Inserted |
| L220: | Replace Findings with Results | Replaced |
| Figures 1,2,3,4 | The photos in figures 1, 2, 3 and 4 must be combined on a single plate. | Thanks editor, which I fully accept and corrected. |
| Figures 5,6 | Figure 5 and 6: Improve the quality of these figures, remove the background, make the bars not 3D, look for better contrast colors, and name the axes. | Thank you for your comments, which I fully acknowledge. I have revised the figures as per your suggestions, ensuring that each one is saved individually as a TIFF or EPS file. |
| L134 | It is important to add the age of the bulls and their weight, as well as the ivermectin dose given in a table. | Prepared in table |
|  | - It is important to include in the discussion the effect of having done the experiment in the dry season where the abundance and diversity of invertebrates is much lower. How did this affect the results? If it had been done during the rainy season, would they have been different? | Dear Reviewer, We appreciate your concern. During the rainy season, increased water runoff and erosion can wash away dung, limiting invertebrates' interaction with it and hindering decomposition. This disruption affects the natural fermentation process, making it challenging to accurately evaluate the role of invertebrates in soil fertility. Conversely, the dry season offers a more stable environment for dung accumulation and invertebrate activity, providing a clearer insight into their impact. |
|  | The methodology they used to quantify and monitor the decomposition rate of excrement is not clear; you should detail it better in the Data Analysis section. | Thank you for your valuable comments. I have carefully revised this section to align with the reviewers' feedback. |
| - L224-225: | How were all these factors measured? with what instruments? with what criteria? in what units? This should be mentioned in detail in the methodology section. Review and include. | Dung beetles and invertebrates were collected using hand-sorting and pitfall traps, identified with a stereomicroscope and taxonomic keys, and abundance recorded per dung pat. Dung decomposition was assessed by weight loss over time, measured in grams per day using a digital balance. |
| - L227: | What characteristics of the germinated seeds were noted? | Ivermectin does not directly impact seeds but affects invertebrates vital for soil fertility and seed growth. Any decline in seed germination or plant development in ivermectin-treated areas is likely an indirect consequence of its effect on soil-dwelling invertebrates, which are essential for nutrient cycling, soil aeration, and seed dispersal. |
|  | Table 1: Grouping all beetles into a single category is very poor. There are many families associated with excrement with very different functions and roles such as coprophages, saprophages and predators. Putting them all in a single group does not allow us to understand their role in the process and how their interaction can be positive or negative. | Dear Reviewer, We appreciate your concern regarding the classification of beetles into a single category. However, our experiment primarily focused on evaluating the impact of ivermectin exposure on beetle survival rather than analyzing the functional roles of different beetle families in dung decomposition. Our study specifically aimed to assess beetle abundance under ivermectin exposure and its subsequent effect on the decomposition process. |
| L440-441: | The toxic effect of ivermectin on seeds must be studied in detail with specific experiments to evaluate this relationship. | Ivermectin does not directly impact seeds but instead affects invertebrates that are vital for soil fertility and seed growth. Any decline in seed germination or plant development in ivermectin-treated environments is likely an indirect result of its effect on soil-dwelling invertebrates, which are essential for nutrient cycling, soil aeration, and seed dispersal. |
